# Extracellular vesicles from neural progenitor cells promote functional recovery after stroke in mice with pharmacological inhibition of neurogenesis

**DOI:** 10.1038/s41420-023-01561-4

**Published:** 2023-07-28

**Authors:** Aura N. Campero-Romero, Fernando H. Real, Ricardo A. Santana-Martínez, Tonatiuh Molina-Villa, Cristina Aranda, Emmanuel Ríos-Castro, Luis B. Tovar-y-Romo

**Affiliations:** 1 Department of Molecular Neuropathology, Instituto de Fisiología Celular, Universidad Nacional Autónoma de México, Ciudad de México, Mexico; 2 Department of Cellular and Developmental Biology, Instituto de Fisiología Celular, Universidad Nacional Autónoma de México, Ciudad de México, México; 3 Unidad de Genómica, Proteómica y Metabolómica, LaNSE, Cinvestav-IPN, Ciudad de México, México

**Keywords:** Stroke, Stroke, Cell death in the nervous system, Mechanisms of disease

## Abstract

Neural progenitor cells (NPCs) of the subventricular zone proliferate in response to ischemic stroke in the adult mouse brain. Newly generated cells have been considered to influence recovery following a stroke. However, the mechanism underlying such protection is a matter of active study since it has been thought that proliferating NPCs mediate their protective effects by secreting soluble factors that promote recovery rather than neuronal replacement in the ischemic penumbra. We tested the hypothesis that this mechanism is mediated by the secretion of multimolecular complexes in extracellular vesicles (EVs). We found that the molecular influence of oxygen and glucose-deprived (OGD) NPCs-derived EVs is very limited in improving overt neurological alterations caused by stroke compared to our recently reported astrocyte-derived EVs. However, when we inhibited the ischemia-triggered proliferation of NPCs with the chronic administration of the DNA synthesis inhibitor Ara-C, the effect of NPC-derived EVs became evident, suggesting that the endogenous protection exerted by the proliferation of NPC is mainly carried out through a mechanism that involves the intercellular communication mediated by EVs. We analyzed the proteomic content of NPC-derived EVs cargo with label-free relative abundance mass spectrometry and identified several molecular mediators of neuronal recovery within these vesicles. Our findings indicate that NPC-derived EVs are protective against the ischemic cascade activated by stroke and, thus, hold significant therapeutic potential.

## Introduction

For a very long time, the use of neural stem cells has been regarded as a potential treatment for neurological pathologies such as amyotrophic lateral sclerosis, spinal cord injury, Parkinson’s disease, Alzheimer’s disease, multiple sclerosis, and stroke due to their intrinsic capacity for cell replacement as well as their paracrine effects [1]. In experimental stroke, it is well characterized that an expansion of neural progenitor cells (NPC) of the subventricular zone (SVZ) takes place following the insult, and the newly generated cells migrate to the lesioned tissue [2–4]. It is also known that about 80% of the newly generated cells that express neuronal markers disappear by six weeks post-ischemia, arguably due to the prevailing improper milieu and neuroinflammation [4]. Although, under certain conditions, cells formed after stroke seem to be able to develop into mature neurons that escape apoptotic cell death [5]. However, this phenomenon might be limited to occurring in rodents, as the basis of neurogenesis in the human adult brain remains controversial [6–8].

Nonetheless, ample evidence exists on the reparative effects of NPC in experimental models for CNS diseases by secreting immunomodulatory molecules and growth factors [9, 10]. Several studies, predominantly using NPCs transplants, have shed light on the paracrine mechanism proposed to promote neural recovery of the infarcted brain [11–13]. For instance, human NPCs have been shown to produce and release trophic factors that promote angiogenesis and neuronal repair, like vascular endothelial growth factor (VEGF) [12, 14], which is essential in the recovery of neurons following an ischemic insult by activating both VEGFR1 and VEGFR2 [15, 16].

An alternative mechanism for this protection is that NPCs secrete neuroprotective factors not as soluble molecules but in more complex structures, like extracellular vesicles (EVs). We have recently shown that EV-mediated intercellular communication between astrocytes and neurons promotes a faster neurological recovery following a stroke by enhancing axon outgrowth in the lesioned brain [17]. In this regard, human NPC-derived EVs have been shown to ameliorate neurological deterioration in experimental stroke [18].

Many recent studies have assessed the effects of EVs derived from stem cells that do not necessarily originate from the brain and have described a high potential to improve recovery in stroke [19–25]. In this sense, a recent clinical trial applying mesenchymal stem cells in patients with chronic major stroke found a five-fold elevation of circulating EVs associated with an improved motor function that was not necessarily related to increased circulating trophic factors [26]. However, it is very well described that the cargo content of EVs heavily depends on the originating cell and the environmental conditions at the time of release [27, 28]. Therefore, assessing NPC-derived EVs’ effect is essential to understanding their protective potential.

NPC-derived EVs carry a plethora of signaling and bioactive molecules, including active enzymes [29]. Here, we set out to describe the proteomic content of EVs derived from NPC from the SVZ of the mouse adult brain under basal culturing conditions and following an ischemic challenge induced by oxygen and glucose deprivation (OGD). We describe that these EVs contain significant quantities of proteins involved in biological processes relevant to neuronal maintenance, survival, and recovery after brain injury. We tested their potential to promote neuronal survival in vitro and whether administering these vesicles to mice subjected to experimental stroke would improve their neurological recovery. We found a critical improvement in neurological performance only in animals subjected to the chronic inhibition of the proliferation of neuronal progenitors. The results presented here are valuable to understand the refined molecular processes mediated by proliferating NPCs that help the brain recover after a major insult such as an ischemic stroke.

## Results

### EVs isolation and characterization from adult lateral ventricle NPCs

**Fig. 1:**
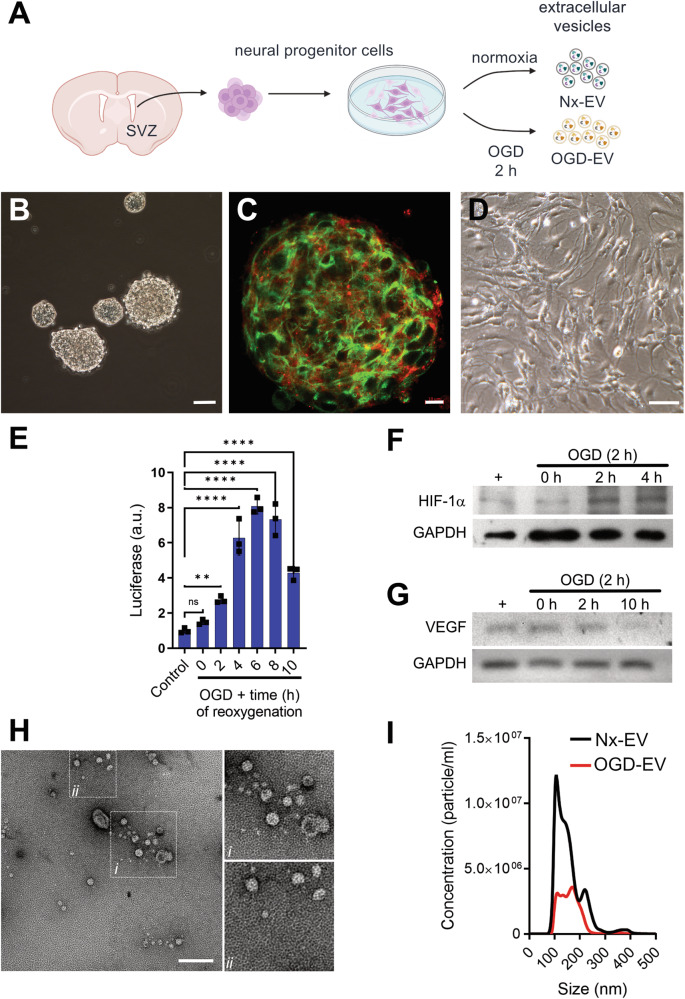
NPCs in culture readily release EVs. **A** Schematic representation of EV collection from primary NPCs in culture. **B** Neurospheres grown in vitro for 4 days. Bar equals 50 µm. **C** Expression of neuronal progenitor markers nestin (green) and doublecortin (red). Bar equals 10 µm. **D** NPC’s growing in monolayers in vitro. Bar equals 20 µm. **E** OGD-induced luciferase reporter gene expression at different time points following reoxygenation. **F** Expression of HIF-1 protein at 2 and 4 h after OGD in NPCs. **G** Expression of VEGF mRNA at 2 and 10 h after OGD in NPCs. **H** Transmission electron microscopy micrographs of EVs isolated from NPCs cultures for 24 h. Vesicles were visualized by negative staining with uranyl formate on copper/carbon-coated grids. Bar equals 200 nm. **I** Size distribution of particles in suspensions by nanoparticle tracking analysis of EVs isolated under normoxia for 48 h and after a 2-h OGD stimulus and 42-h recovery.

Freshly isolated adult CD1 mouse NSCs from the lateral ventricle typically grow on non-adhesive plates to form neurospheres (Fig. 1A, B). In these clusters, cells expressed the markers for neuronal precursors nestin and doublecortin (Fig. 1C). Secondary cultures derived from primary neurospheres were grown in monolayers to harvest EVs from the conditioned medium (Fig. 1D). These cells were subjected to oxygen and glucose deprivation (OGD) for 2 h and then, fresh media with glucose and oxygen was reintroduced for 22 h. We determined NPCs’ response to the OGD challenge by assessing the activity of firefly luciferase transfected in a vector containing three hypoxia response elements from the phosphoglycerate kinase 1 promoter. We determined that under our experimental conditions, hypoxia-induced gene expression peaked at 6 h (Fig. 1E). We also observed the increased synthesis of HIF-1α protein (Fig. 1F, Supplemental material shows full uncropped WB), and VEGF mRNA (Fig. 1G) that were elevated 2 h after the stimulus.

After an incubation of 24 h, the conditioned medium was collected, and EVs were harvested by ultracentrifugation. We obtained a homogeneous population of small vesicles with the typical shape of exosomes in both conditions, as revealed by transmission electron microscopy (Fig. 1H). The sizes and quantities of the isolated EVs were assessed by nanotracking analyses (NTA) with a peak size in the range of exosomal dimensions in both conditions; normoxia 156.0 ± 7.7 nm and OGD at 164.9 ± 16.6 nm (mean ± SD) (Fig. 1I). A few EVs with larger dimensions are also present in the preparation.

### Protective effects of EVs on OGD-induced neuronal death

**Fig. 2:**
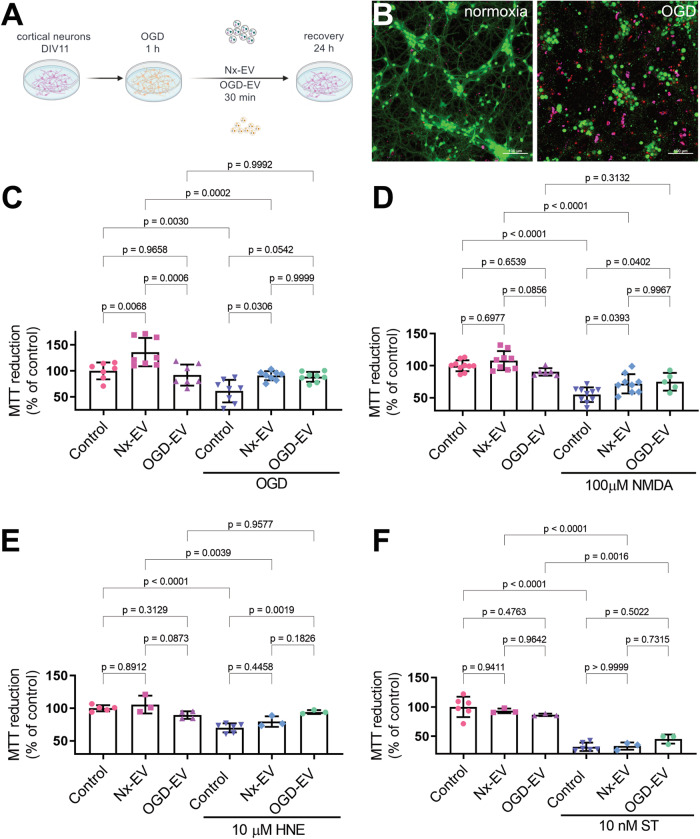
NPC-EV protect cortical neurons against ischemic death. **A** Schematic representation of the experimental design to expose cortical neurons to OGD-induced injury and administration of NPC-EVs. **B** Representative images of cortical neurons under control conditions (right) and following 1 h OGD (left). CytoCalcein stain labels alive neurons (green), while dead cells are marked by the nuclear staining of 7-AAD (red) for necrotic cells or apopxin (magenta) for apoptotic cells. Bar equals 100 µm. Assessment of neuronal viability by MTT reduction of cultures exposed to 1 h OGD (**C**), 100 µM NMDA (**D**), 10 µM HNE (**E**), or 10 nM ST (**F**), supplemented with Nx-EV or OGD-EV. Graphs show the mean ± S.D. with individual experiments plotted to show the number of independent experiments and data distribution per condition. ANOVA followed by Tukey’s post hoc.

Several studies have reported that NPC-derived EVs can potentially protect against neuronal damage in stroke [30–32]. We set out to determine whether the vesicles produced by NPCs in culture under normoxic (Nx-EV) and OGD (OGD-EV) conditions would influence neuronal survival following a mild ischemia/reperfusion challenge mimicked by 1 h OGD followed by glucose and oxygen reintroduction (recovery). After 30 min of 1 h-exposure to OGD, primary cortical neurons were administered with a suspension of EVs equivalent to 800 ng/mL of protein content, determined by the Lowry method quantifications (Fig. 2A, B). Cellular metabolism assessed by MTT reduction was used to proxy neuronal survival at 24 h after the OGD exposure. OGD caused a decrease to 61.1 ± 21.7% (*p* = 0.003, compared to control) of baseline MTT reduction in control conditions (i.e., no EVs added), whereas the neurons cultured with NPC-EVs show significant protection from this metabolic damage with 90.8 ± 8.8% (*p* = 0.03, compared to OGD alone) viability for Nx-EV and 88.6 ± 9.6% (*p* = 0.054, compared to OGD alone) for OGD-EV (Fig. 2C).

Ischemic damage triggers a series of molecular mechanisms that eventually drive neurons to die. The ischemic cascade encompasses excitotoxicity, oxidative stress, and apoptosis, among several other molecular mechanisms. We set out to determine which of these three mechanisms would be relieved by NPC-EVs. For this, we exposed individual cultures to N-methyl-D-aspartate (NMDA), 4-hydroxynonenal (HNE), and staurosporine (ST) to mimic the processes mentioned above. Neurons exposed to 10 µM NMDA to produce excitotoxicity reduced their cellular viability to 54.8 ± 11.3% (*p* < 0.0001) of control, and Nx-EVs were able to increase that value to 71.9 ± 15.0% (*p* = 0.03, compared to NMDA alone), whereas OGD-EV did to 73.1 ± 15.9% (*p* = 0.04, compared to NMDA alone) (Fig. 2D). Exposure of neuronal cultures to 10 µM HNE to induce oxidative damage reduced cell viability to 69.9 ± 6.8% (*p* < 0.0001 compared to control, Fig. 2E). In this case, Nx-EV did not induce an apparent recovery of neurons (79.5 ± 8.0%, *p* = 0.445 compared to HNE alone), but OGD-EV did (94.2 ± 2.9%, *p* = 0.0019 compared to HNE alone). Finally, we tested whether adding NPC-EV would halt apoptosis induced by the kinase inhibitor ST. This treatment induced a dramatic reduction of neuronal viability (33.1 ± 7.1%, *p* < 0.0001 compared to control; Fig. 2E) that was not blocked by Nx-EV (31.1 ± 6.1%, *p* > 0.999 compared to ST alone) or OGD-EV (45.0 ± 7.7%, *p* = 0.502, compared to ST alone) (Fig. 2F).

These results suggest that NPC-EV have the potential to counteract OGD-induced neuronal and some of the ischemic cascade processes like excitotoxicity and oxidative stress.

### NPC-derived Nx-EV promote post-ischemic neurological recovery in animals with neurogenesis ablation

**Fig. 3:**
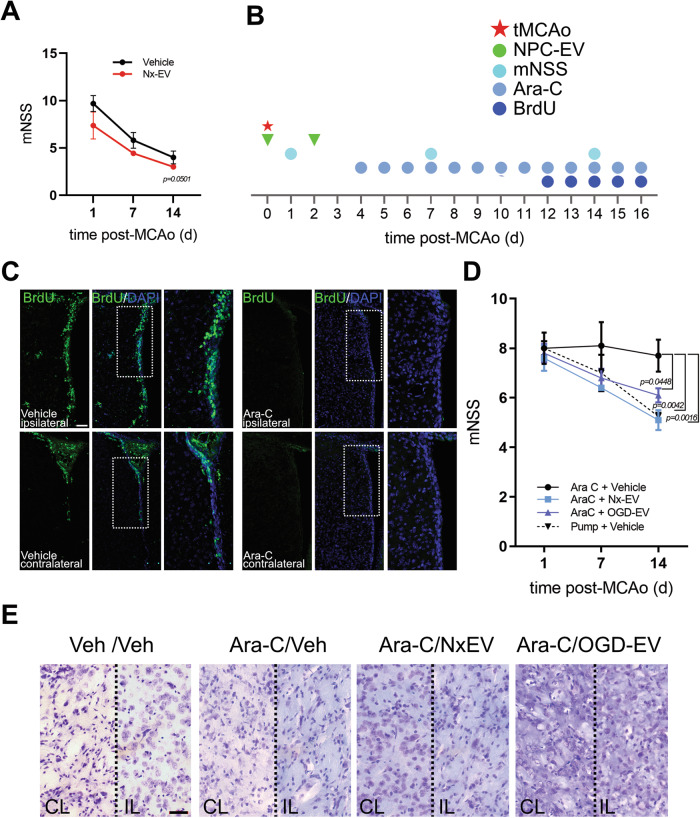
NPCs influence post-stroke recovery through EVs. **A** Modified neurological severity score (mNSS) assessed over time in animals subjected to tMCAo and administered with vehicle or Nx-EV (*n* = 4 per group). The graph shows the mean ± S.E.M. *p* = 0.0501, unpaired *t*-test. **B** Time scale of experimental procedures for endogenous NPC proliferation inhibition after stroke, and the administration of exogenous NPC-EVs. **C** Representative micrographs of the subventricular zone of rats subjected to tMCAo implanted with an osmotic minipump that delivered vehicle (left panels) or Ara-C (right panels). The production of new cells was labeled with BrdU (green), and cell nuclei were dyed with DAPI (blue). Scale bar equals 50 µm. **D** mNSS over time in animals subjected to tMCAo and the inhibition of neurogenesis through the continuous administration of Ara-**C** administered with Nx-EV or OGD-EV at 30 min and 48 h post-stroke. (*n* = 5 per group in the animals chronically infused with Ara-C, and *n* = 4 in animals implanted with a vehicle-filled osmotic minipump). The graph shows the mean ± S.E.M., ordinary one-way ANOVA followed by Dunnett post hoc. **E** Representative micrographs of Nissl stainings of the striatum in the infarct core (IL; ipsilateral) or contralateral (CL) side 16 d after stroke, in mice that implanted with osmotic minipumps filled with vehicle (far left) or Ara-C (three images to the right) with or without NPC-EV administered i.c.v. Scale bar equals 20 µm.

We sought to determine whether the administration of NPC-EV in vivo would protect against ischemic damage. For this, mice subjected to 40 min transient middle cerebral artery occlusion (tMCAo) were injected i.c.v., 30 min after the beginning of reperfusion, with a suspension of Nx-EV with a total protein content equal to 800 ng. A second administration was performed two days after tMCAo. These animals were evaluated with a modified neurological severity score (mNSS) which combines motor, sensor, reflex, and balance tests and overall health status described in Table 1, at 1, 7, and 14 d post-stroke. In these animals, there was a clear improvement in neurological function, corresponding to the spontaneous recovery that has been amply described to occur in stroke in human patients and animal models [17, 33] (Fig. 3A). However, the administration of Nx-EV did not increase the recovery of the animals, and the group administered with these vesicles was not different from the control group that received i.c.v. vehicle only (Fig. 3A).

**Table 1:** Items evaluated in the modified neurological severity score (mNSS) and their respective value.

Overall health status			
Assessment			Score
Hair		Normal condition	0
		Lack of grooming, piloerection, and dirt on the fur around the nose and eyes	1
		Lack of grooming, piloerection, and dirty fur, extending beyond the nose and eyes	2
Eyes		Open and clear	0
		Open with milky mucus	1
		Open with dark mucus	2
		Eyes clotted (one or both)	3
		Closed	4
Spontaneous activity		The mouse is alert and actively explores the arena.	0
		The mouse is alert but remains still.	1
		The mouse slowly starts exploring and stops repeatedly, OR, the mouse is listless and does not explore the arena.	2
		The mouse is lethargic and barely moves during 60 s.	3
		No spontaneous movements	4

Focal deficits			
Objective	Assessment		Score
Body symmetry	The mouse is placed in an open observation field for undisturbed resting behavior	Normal body posture, trunk elevated from the bench, forelimbs and hindlimbs leaning beneath the body, and straight tail.	0
		The body leans on one side, the fore and hindlimbs beneath the body, and the tail slightly bent.	1
		The body leans on one side, the fore and hindlimbs stretched out, and the tail slightly bent.	2
		The body leans on one side with the tail bent.	3
		The mouse is fully tilted towards the contralesioned side.	4
Gait	The mouse is placed in an open observation field for undisturbed movement behavior	The mouse has a flexible, symmetric, and quick gait.	0
		The mouse is stiff and walks humpbacked, slower at a slower pace.	1
		The mouse shows limping with asymmetric movements.	2
		The more severely limps, drifting and falling with an apparent deficiency in the stride.	3
		The mouse does not spontaneously walk unless it is gently pushed and cannot take more than three steps.	4
Climbing	The mouse is placed in the center of a grid at a 45° angle.	The mouse quickly climbs upwards.	0
		The mouse can climb slowly, with apparent limb weakness.	1
		The mouse holds on to the grid and does not move.	2
		The mouse is unable to stand on the grid and moves downwards.	3
		The mouse falls when put on the grid.	4
Circling behavior	The mouse is placed in an open observation field.	Circling behavior is absent. The mouse equally turns left and right.	0
		The mouse turns preferentially towards the ipsilateral side.	1
		The mouse makes a full circle towards the ipsilateral side.	2
		The mouse keeps constantly rotating towards the ipsilateral side.	3
		The mouse does not move.	4
Forelimb symmetry	The mouse is suspended by the tail.	The mouse extends both forelimbs toward the floor. It keeps moving actively while suspended.	0
		The mouse does not extend the contralateral forelimb entirely.	1
		The mouse shrugs the contralateral forelimb and maintains a tilted position towards the ipsilateral side.	2
		The contralateral forelimb is fully shrugged.	3
		The mouse does not move while suspended	4
Compulsory circling	While the mouse is suspended by the tail, it is allowed to rest the forepaws on the surface of an evaluation table. This position reveals the presence of contralateral limb palsy. In this handstand position, limb weakness is displayed by a circling behavior when the animal attempts forward motion	The mouse displays normal extension of both forelimbs.	0
		The mouse extends both forelimbs and rotates preferentially towards the ipsilateral side.	1
		The mouse circles towards the ipsilateral side.	2
		The mouse pivots to the ipsilateral side but does not rotate in a full circle and eventually falls on its side.	3
		The mouse does not move.	4
Paw grip endurance	The mouse is placed on a grid while it is held by the tail.	The mouse firmly holds on to the grid with its forepaws and tries to place the hind paws onto the grid by pulling them under the body.	0
		The mouse accesses the grid but with less strength. A slight pull breaks the grip of the forepaws.	1
		The mouse cannot hold on to the grid with the lesioned forepaw.	2
		The mouse cannot hold onto the grid at all.	3

This result overtly contrasts our previous finding that EVs derived from astrocytes facilitate spontaneous recovery in the rat after tMCAo [17]. Therefore we hypothesized that if NPC-EV had any neuroprotective effect, this was being masked by the endogenous process already happening in the in vivo model, so we set out to determine whether a possible neuroprotective effect would become clearer under conditions that impede the proliferation of endogenous NPCs after stroke. For this, four days after stroke, mice were implanted with an osmotic minipump that delivered the DNA synthesis inhibitor cytosine-D-arabinofuranoside (4%, Ara-C) at a rate of 0.25 µl/h for 16 d (Fig. 3B). Ara-C has been previously reported to inhibit cell proliferation in the mouse SVZ [34]. We confirmed that this experimental protocol effectively inhibited the proliferation of NPCs following stroke by BrdU labeling of the SVZ at 16 d (Fig. 3D). Following tMCAo, we observed an increase of BrdU^+^ cells in the lateral walls of the SVZ, as previously reported elsewhere, both in the ipsilateral and contralateral sides of the infarct (Fig. 3D). BrdU^+^ cells were largely absent in the animals treated with Ara-C.

The spontaneous recovery presented by the animals subjected to tMCAo with vehicle alone (Fig. 3A) was lost in the animals chronically infused with Ara-C (Fig. 3D), as they did not recover over time. This sustained neurological impairment was not artifactually due to the subcutaneous minipump’s presence, as animals implanted with a vehicle-filled pump could recover spontaneously (pump + vehicle group in Fig. 3D). Remarkably, the administration of Nx-EV or OGD-EV, twice at 30 min and 48 h post-stroke, promoted the neurological recovery of the treated animals to the same level seen in those that did not receive Ara-C (Fig. 3D).

The histological alterations of the infarct core in the striatum are not very evident by Nissl staining 16 d post-stroke (Fig. 3E), as has been reported before [35, 36].

This result strongly suggests that the endogenous occurrence of NPC proliferation after stroke is essential for spontaneous recovery through a mechanism that involves intercellular communication via EVs. However, the contribution of NPC-EV is limited and does not potentiate the effect that naturally occurs after a stroke in this preclinical model.

### Characterization of the proteomic content of NPC-shed EVs

The EV protein content directly reflects the internal protein production of the parent cell and, thus, changes after external stimulation. We, therefore, set out to investigate the differences in protein content following OGD. For this, we ran a label-free relative abundance characterization by mass spectrometry.

**Fig. 4:**
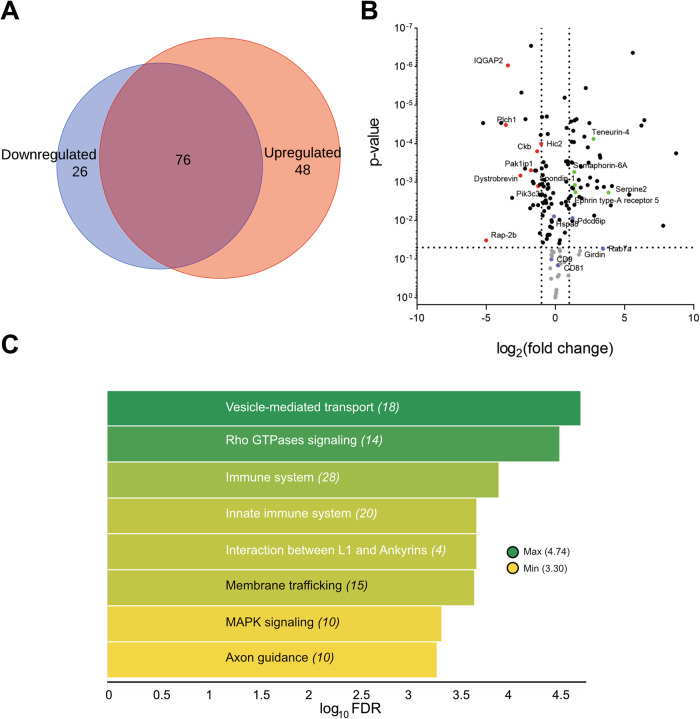
Proteomic profiling of NPC-derived EV under OGD. **A** Venn diagram shows the proportion of proteins in EV released by NPCs that are selectively down or upregulated after an OGD stimulus. **B** Volcano plot shows individual proteins that are significantly (*p* < 0.05) down- or upregulated (fold change ≥ 1) after an OGD stimulus. **C** Heatmap of the Reactome pathway analysis summarizing the GO biological processes represented in the proteomic content of EVs released from NPC after OGD. The number indicated in parenthesis for each biological process corresponds to the observed gene count. The false discovery rate (FDR) value was converted to log_10_ for generating the color-pondered heatmap. The heatmap was generated with TIBCO Spotfire.

We obtained 150 hits, including several exosomal markers such as CD9, CD81, Pdcd6ip, and Hspa8 (Fig. 4A, B). Seventy-six identified proteins were present both under normoxia and OGD, 26 proteins were found to be downregulated in OGD relative to normoxia, and 49 proteins were upregulated.

We used the Reactome pathways module to identify putative pathways of protein cargo in NPC-EV (Fig. 4C). Protein enrichment analyses show exosome biogenesis and transport pathways, such as vesicle-mediated transport, signaling by Rho GTPases, the interaction between L1 and Ankyrins, and membrane trafficking, that go along with the role of EV in communicating signaling molecules among cells.

We also found several NPC markers in our proteomic analyses; among those are: the neuron-specific isozyme phospholipase C PLC(eta)2 [37], Teneurin-4, involved in neuronal development and cell adhesion required for the differentiation of oligodendrocytes [38–40], Nedd-4 that participates in neurite development [41].

Of particular relevance to this study, we found that axon guidance, one of the several endogenous mechanisms for recovery after brain ischemia [16], was among the pathways overrepresented in the Reactome pathways, with the appearance of proteins like IQGAP2, Trio, Teneurin-4, Semaphorin 6A, Serpine2, Spondin-1, Ephrin type receptor 5.

A complete list of the identified proteins can be found in the ProteomeXchange Consortium via the PRIDE [42] partner repository with the dataset identifier PXD033915.

## Discussion

This study shows that NPCs’ contribution to recovery following stroke is mainly driven by a mechanism of intercellular communication mediated through EVs. Several studies have revealed the ability of neuronal precursors to survive and proliferate under ischemic conditions [43]. In preclinical models, these precursors can differentiate and migrate within the ischemic environment, making them a promising translatable therapeutic target for stroke and other brain injuries [44]. However, as discussed above, a likely mechanism for protecting neurons after ischemic stress is synthesizing and releasing signaling molecules with trophic activity.

The trophic effects of neuronal progenitors on neurons involve the production of neurotrophic factors, signaling molecules, cytokines, chemokines, metabolites, and bioactive lipids, which promote neuronal survival, differentiation, and maturation [45]. These factors can also support the formation of synaptic connections, increase the neuronal ability to survive environmental stresses and protect neurons from damage, such as excitotoxicity, hypoxia, and oxidative stress [46]. In addition, neuronal progenitors can secrete cytokines, which can induce the growth of healthy neuronal networks and regulate the expression of specific genes involved in the development of neurons [47]. The trophic effects of NPCs on neurons can thus provide a means to repair damaged neural circuits and restore normal neural function [48].

In the later years, we have witnessed an ever-increasing interest in the field of neuronal recovery post-stroke using EVs. Several examples in the literature show their potential to increase recovery; EVs isolated from NPCs in combination with vesicles from endothelial progenitor cells facilitate the protection of endothelial functions by regulating the trophic activity of brain-derived neurotrophic factor (BDNF) by delivering miR-126 and miR-210 [32] . We saw here that NPC-EVs could rescue cultured neurons from death-inducing stimuli relevant to the ischemic cascade. Other studies employing EVs derived from human induced pluripotent stem cells (iPSC-NPCs) have reached similar conclusions [31, 49]. Furthermore, the effect of EVs from human nerve and glial cells has been shown to induce neuroprotection against OGD-induced neuronal damage [50].

Previous reports have indicated that several molecular mechanisms are potentially involved in regulating neuroprotection, for example, activating the Jak2/Stat3 pathway downstream endothelin-1 [51], Sonic hedgehog [52], Wnt/β-catenin [53], p27Kip1 [54], mitogen and stress-activated kinases 1/2 [55], and PI3-K/Akt [56]. We, therefore, investigated the proteomic content of NPC-EVs after an ODG challenge. Some of the proteins we identified in our analysis hold attractive molecular potential for the recovery effects reported in the present study; for example, IQGAP-2 is required for axon elongation, axon outgrowth, and appropriate growth cone morphology in hippocampal neurons [57]. Trio, a scaffolding protein containing Rac and Rho GEF domains, is a key regulator of neuronal migration, axon outgrowth, axon guidance, and synaptogenesis by activating the RAC1 GTPase modulating actin cytoskeleton remodeling [58, 59]. Rab7, a late endosome membrane-associated G protein involved in vesicular trafficking, has been implicated in regulating protein synthesis in the axon terminals [60]. Thus, it could be speculated that EVs carry within molecular complexes that facilitate axon repair. Another target protein in our proteomic analysis is semaphorin 6a, a protein known to influence axon guidance [61]. Other proteins like ephrin type-A receptor 5 is homologous to proteins involved in the vascular remodeling after stroke [62].

This proteomic analysis provides insight into the possible biological activity of NPC-EVs and suggests that axon guidance might contribute to recovery after brain ischemia. However, the present analysis is limited to the capabilities of the mass spectrometry technique and the relative abundance of proteins in EVs, biasing only for the most represented ones. Several additional parameters for the characterization of the molecular cargo, including analyzing the regulatory RNA content, are needed to use this information in the mechanistic context of brain repair after stroke [27].

In this study, we found that the extent of the protective effect conferred by factors in NPC-EVs is moderate, and similarly to what has been previously reported, it does not necessarily impact the infarct volume [18]; this could be partially explained by the relatively low number of cells contributing to the protective effect. Also, the neurogenesis process in rodents peaks around one to two weeks after the stroke [63], and by then, the establishment of the damage might be factually irreversible. Nonetheless, our findings harbor significant translational applicability; given that the occurrence of neurogenesis in the human adult brain is still debatable, our study suggests that the potential of NPCs in the recovery of neurons damaged after a stroke can still be instigated if the proper action is taken to deliver NPC-EVs produced exogenously.

Also, it is worth noticing that since the second administration of NPC-EV was carried out at 48 h and neurogenesis is an event that in rodents occurs several days after the stroke, an extended time window for therapeutic intervention is possible.

In conclusion, this study shows that NPC proliferation in response to ischemia is part of an endogenous mechanism of brain recovery with a translatable therapeutic potential conferred by the capability of these cells to synthesize and release in EVs molecular factors that could be delivered to the injured brain with the appropriate clinical interventions. This alternative therapeutic approach is worth exploring in the setting of stroke, for which minimal options currently exist.

## Methods

### Animals

This study used young 8-week-old wild-type CD1 mice subjected to MCAO as described below. Mice were bred at the Animal Facility of IFC-UNAM, certified by the Secretariat of Agriculture and Rural Development (SADER-Mexico). Animals were housed in individual cages in a 12-h light/12-h dark cycle with food and water ad libitum. Mice were killed at 16 days post-stroke. All experimental procedures were conducted under the current Mexican law for the use and care of laboratory animals (NOM-062-ZOO-1999) with the Institutional Animal Care and Use Committee approval (CICUAL-IFC-LTR93-16). Experiments are reported in compliance with the Updated Animal Research: Reporting In Vivo Experiments (ARRIVE 2.0) guidelines [64].

### Study design

For the in vivo experiments, the sample size used was determined a priori based on pilot experiments. Considering a medium Cohen’s d effect size >0.3, statistical b power of 0.8, and significance of 0.05, we determined that *n* = 5 would allow us to reject the null hypothesis with 95% confidence. The mortality rate was assumed to be 0.5 based on pilot experiments. The inclusion criteria consider the analysis of experiments that recapitulate complete ischemia/reperfusion without brain hemorrhagia. An experiment met the inclusion criteria when there was a reduction of blood perfusion below 50% of baseline, which roughly corresponds to the effect of occluding the common carotid artery, reperfusion above 50% baseline within 10 min, total occlusion time of 40 min, absence of subarachnoid or intraparenchymal hemorrhages, and survival for 24 h after stroke. For humane reasons, experiments were terminated when animals presented with hemiplegia or generalized weakness that made them unable to eat or drink autonomously within 24 h of stroke; those animals were considered dead before 24 h.

### Adult neural progenitor cells

NPCs were obtained from the subventricular zone of 8-week-old male CD1 mice. Animals were killed by cervical dislocation followed by decapitation. The brains were collected in ice-cold Hank’s balanced salt solution (HBSS, Invitrogen), and the SVZ of the lateral ventricles was dissected under a stereoscopic microscope. Tissue from four mice was pooled for each culture. Dissected tissues were minced and incubated with TrypLE Express (Invitrogen) for 5 min at 37 °C. Then, an equal volume of Neurobasal medium (Gibco) was added, and the tissue was mechanically disaggregated with a fire-polished Pasteur pipet tip. The cell suspension was passed through a 40 µm-pore cell strainer and subsequently centrifugated at 300 × *g* for 3 min. The pellet was resuspended in Neurobasal + B27 supplemented with 20 ng/ml recombinant epidermal growth factor (EGF; Preprotech, 400-25) and 10 ng/ml recombinant basic fibroblast growth factor (bFGF; Preprotech, 450-33). After 3–4 DIV, neurospheres began to grow in suspension; when spheres reached 150-200 µm in diameter (5–7 DIV), they were dissociated by enzymatic digestion with TrypLE Express at 37 °C for 3 min, then the number of viable cells was estimated by trypan blue exclusion, and cells were plated at a density of 8000 cells/cm^2^ in poly-D-lysine/laminin-coated T75 flasks. NPCs were used at passage ≤ 5 in all experiments.

### Oxygen and glucose deprivation

Under some experimental conditions, the cells were subjected to oxygen and glucose deprivation (OGD). For this, cultured cells underwent a complete medium change to DMEM:F12 without glucose and pyruvate (GIBCO) and were incubated inside a hypoxic chamber (StemCell Technologies, Cambridge, MA) with a 100% N_2_ atmosphere for 2 h at 37 °C. Following this period, the cells were put back in Neurobasal media supplemented with B27 and incubated under normoxic conditions for 22 h.

The molecular response to OGD in NPCs was corroborated by analyzing Western blot’s expression of the hypoxia-inducible factor α. For this, 10 µg of whole cell lysates were resolved by 7.5% PAGE and transferred to a PVDF membrane that was probed with a mouse primary monoclonal antibody (1:200, Santa Cruz; sc-13515) and an HRP-conjugated goat anti-mouse antibody (1:5000, GeneTex; GTX213111-01) Following incubation with the Immobilon forte substrate (EMD Millipore), the blot was exposed to a photographic film (Kodak). In addition, the expression of VEGF mRNA was assessed by RT-PCR. Total RNA was extracted from NPC with TRIzol (Invitrogen, Carlsbad, CA) for this. An amount of 1 µg was reverse transcribed to cDNA, and an aliquot of 1 µl of cDNA was used as a template for RT-PCR using an Applied Biosystems system with the following parameters: VEGF forward primer: 5′ GGCCTCCGAAACCATGAACT3′, reverse primer - 5′ GTCCACCAGGGTCTCAATCG 3′ that amplify a product of 141 bp; GAPDH forward primer 5′-GCATCTTCTTGTGCAGTGCC-3′ and reverse primer 5′-GATCTCGCTCCTGGAAGATGG-3′ that amplify a product of 278 bp. Thermocycling conditions were melting at 95 °C for 30 s; anneal at 63.5 °C (VEGF 27 cycles) for 45 s, or 57 °C (GAPDH 25 cycles) for 30 s; extension at 72 °C for 30 s. The PCR products were analyzed by electrophoresis in a 2% agarose gel. The relative RNA amount was calculated by densitometry using Image J.

### Immunofluorescence

Neurospheres were allowed to sediment on poly-D-lysine/laminin-coated coverslips. Cells were fixed with ice-cold paraformaldehyde (4%, w/v) for 20 min, then permeabilized with 0.25% Triton X-100 for 5 min, blocked with 5% normal goat serum, and incubated overnight at 4 °C with anti-nestin (1:500, Thermo Scientific, PA5-17428) and anti-doublecortin (1:1000, Thermo Scientific, 2Q178) antibodies. Then, cells were incubated with Alexa Fluor 488-conjugated anti-rabbit (1:500, Life technologies, A11008) and Alexa Fluor 546-conjugated anti-mouse (1:500, Life technologies, A11003) antibodies at RT for 1 h, and the nuclei were counterstained for 5 min with 4′,6-diamidino-2-phenylindole (DAPI). Immunofluorescence was visualized using a Zeiss LSM 800 II Microscope.

### Luciferase assay

Following the manufacturer instructions, NPCs were transfected with HRE-luciferase and pEGFP-N1 plasmids with the mouse NSC nucleofector kit (Lonza, Basel, Switzerland). HRE-luciferase was a gift from Navdeep Chandel (Addgene plasmid #26731; RRID: Addgene_26731) [65], and pEGFP-N1 was a gift from Félix Recillas (IFC-UNAM). Forty-eight hours post-transfection, the cell cultures were subjected to OGD for 2 h. They were lysed, and luciferase activity was determined in a luminometer (Turner Biosystems, Promega) with the luciferase Assay System (Promega, Madison, WI) at different time points after recovery. The number of GFP-positive cells was used to normalize for transfection efficiency.

### EV isolation and quantitation

The conditioned media of NPCs cultured in monolayers was collected after 12 h, then cell debris and apoptotic bodies were removed by centrifugation at 500 × *g* at 10 min. Conditioned media was then filtered with a 0.2 µm pore filter and subsequently ultracentrifuged at 50,000 × *g* for 30 min; the supernatant was further ultracentrifuged at 100,000 × *g* for 4 h (4 °C). The isolated EV pellets were resuspended in 100 μl PBS and stored at −80 °C until used. EV size, distribution, and concentrations were determined as previously reported [17] using an NS300 NanoSight nanoparticle tracking analysis system (Malvern Instruments). Data were binned and plotted as a continuous histogram.

### Transmission electron microscopy

Two µL of EV suspension were loaded onto glow-discharged 400 mesh copper/carbon-coated grids and left to settle for 5 min. After a brief wash with drops of distilled water, the grids were stained in 2% uranyl formate for 1 min and blow-dried on Whatman filter paper. EVs were examined with a JEOL-JEM-1200 Transmission Electron Microscope at an accelerating voltage of 80 keV.

### Cortical neuron cultures

Cortical neuronal cultures were prepared as previously described [15]. E17 rat cortices were isolated, trypsinized, and dissociated in Ca^2+^ and Mg^2+^ free HBSS (Gibco, Carlsbad, CA). Neurons were plated at a density of 1.3 × 10^5^ cells/cm^2^ in polyethyleneimine-coated 24-well plates in Neurobasal medium supplemented with B-27 (Gibco) and 1% antibiotic/antimycotic solution (104 U of penicillin G/ml, 10 mg of streptomycin/ml, and 25 µg of amphotericin B/ml) (Sigma). Cytosine β –D-arabinofuranoside (2.5 µM; Sigma) was added on DIV 3 to prevent the proliferation of astrocytes.

For neuronal death-inducing stimuli, neuronal cultures were incubated on DIV 11 with 10 μM N-methyl-D-aspartate (NMDA) (Tocris) + 1 μM glycine (Sigma), 10 µM 4-hydroxynonenal (Sigma), or 10 nM staurosporine (Sigma) for 24 h. OGD was induced by replacing the conditioned media for glucose and pyruvate-free medium and incubating the cells in a hypoxia chamber (StemCell Technologies, Cambridge, MA) with a 100% N_2_ atmosphere for 1 h, at the end of the OGD period, cells were switched to normal Neurobasal + B27 and placed in a ~21% O_2_ atmosphere. In the indicated experiments, NPC-EVs with a total amount of 800 ng of protein/mL were added to neuronal cultures for 24 h in combination with the death indicing stimuli, or after OGD.

### Assessment of neuronal viability

Neuronal viability was determined as previously described by 3-(4,5-dimethylthiazol-2-yl)-2,5-diphenyltetrazolium bromide (MTT) reduction [15]. Briefly, the neurons were incubated with 0.1 mg/mL MTT for 2 h at 37 °C at the end of the analyzed period. Media was removed, and formazan precipitations were dissolved in 4 mM HCl isopropanol. The absorbance of cell debris-free supernatants was read in a spectrophotometer (Beckman Coulter) with a 570 nm wavelength. Data are presented as a percentage relative to the absorbance of control conditions. In some experimental conditions, we corroborated neuronal viability with the life/dead fluorescence assay (Abcam; 176749, Cambridge, MA) that stain live cells with CytoCalcein AM (green), necrotic cells with 7-aminoactinomycin D (7-AAD; red), and apoptotic cells with apopxin (magenta), following manufacturer’s directions.

### Middle cerebral artery occlusion

Young adult (8-week old; 25 g) CD1 mice were put under anesthesia with xylazine (10 mg/kg i.p., PISA, Guadalajara, Mexico) and maintained anesthetized with ≤1.5% isoflurane (VetOne, Boise, ID) for the duration of the procedure with oxygen as the carrier. Normal ventilation was autonomously maintained. A nylon monofilament with a silicone-dipped tip (602156/602256, Doccol, Sharon, MA) was inserted in the ligated left external carotid artery and intraluminally advanced through the internal carotid artery until it occluded the MCA. The occlusion was kept for 40 min, after which the monofilament was removed. Body temperature was maintained at 37 °C with a heating pad. At the end of the procedure, the neck skin was sutured, and mice were returned to their cages. During the entire experimental procedure, the cerebral blood flow (CBF) was monitored in the territory irrigated by MCA with laser-Doppler flowmetry monitored at the following stereotaxic coordinates; AP -1, L + 5 from Bregma, with a laser-Doppler probe (model 407, Perimed, Järfälla, Sweden) connected to a Periflux System 5010 (Perimed). CBF was continuously monitored with an acquisition interval of 0.3 s using the Perisoft software (Perimed).

### Administration of NPC-derived EVs in vivo

A fixed volume of 2 µl of an exosome suspension in phosphate buffer was administered by intracerebroventricular (i.c.v.) injections in the contralateral lateral ventricle in the corresponding animal groups with the following stereotaxic coordinates: AP + 0.5, L + 1.1 from Bregma and V + 2 from dura matter. i.c.v. administrations were done 30 min after intraluminal filament removal, marking the beginning of reperfusion. A second administration was done on day two post-stroke. Injections were performed with graduated glass microcapillary pipettes (Drummond Scientific Company; Broomall, PA) that were pulled to produce a tip < 50 µm diameter at a flux rate of 0.8 µL/min. The number of EVs administered in each experiment contained equal quantities of total protein in the range of 800 ng.

### Neurogenesis inhibition in vivo

Four percent cytosine-β-D-arabinofuranoside (Ara-C; Sigma) in 0.9% NaCl or vehicle was administered i.c.v. with an Alzet osmotic minipump (model 1002, Durect Co, Cupertino, CA) at a flux rate of 0.25 µl/h for 16 d, starting on day 4 after stroke. Minipumps were connected to an i.c.v. cannula implanted on the skull at stereotaxic coordinates AP + 0.5, L + 1.1 from Bregma, and V + 2 from dura matter. Bromodeoxyuridine (BrdU; 50 mg/kg, Sigma) was injected i.p. daily from day 12 to 16 post-MCAO.

For immunohistological analyses of BrdU^+^ cells, animals were transcardially perfused with 10 mL ice-cold 0.9% NaCl followed by 10 mL ice-cold 4% paraformaldehyde (PFA). The brains were collected and post-fixed in 4% PFA for 24 h and then cryoprotected in 30% sucrose. Whole PFA-fixed brains were cut into 40 µm thick sections in a cryostat to produce ten series of consecutive sections 400 µm apart. Sections were washed in 1X PBS + 0.1% Tween 20 (PBST) for 10 min following antigen retrieval in 50% formamide in 2× saline-sodium citrate buffer (SSC; 300 mM NaCl, 30 mM sodium citrate) for 1 h at 65 °C, washed twice with SSC at RT 10 min each, and incubated with HCl 2 N for 5 min at RT. Next, sections were incubated in 0.1 M boric acid for 10 min and washed for 10 min with PBST twice. Brain sections were blocked with 5% goat serum, 0.5% Triton X-100, 1% BSA, 50 mM glycine in PBST for 2 h at RT, incubated with primary antibody anti-BrdU (1:200; Sigma, SAB4700630-1) in PBST with 10 mM glycine and 1% DMSO overnight at 4 °C and washed thrice with PBST for 10 min each. A secondary anti-mouse antibody conjugated with Alexa Fluor 488 (1:500, Invitrogen, A21202) was incubated in PBST overnight at 4 °C and washed twice with PBST. The sections were incubated with DAPI for 20 min, washed twice with PBST, and mounted on slides with Vectashield mounting medium (Vector Labs, Burlingame, CA). Images were obtained in a Zeiss LSM 800 confocal microscope, and Z-stack images were obtained with FIJI software.

### Neurological evaluation

Animals were evaluated with a battery of neurological tests for body posture and movement control at 1, 7, and 14 days after stroke. The severity of functional deficits was scored by assessing ten items described in Table 1. Two trained observers blinded to the experimental treatment performed all evaluations independently.

### LC-MS analysis

A total of 17 µg of protein per condition were loaded and separated in a single 12% SDS-PAGE lane; then, the gel was stained using a Silver Staining kit (Life Technologies). Each lane was cut into ten fractions, which were enzymatically digested as previously described [66]. The generated tryptic peptides were concentrated to an approximated volume of 15 μL, of which 4.5 μL were loaded and separated on an HSS T3 C18 Column (Waters, Milford, MA); 75 μm × 150 mm, 100 A° pore size, 1.8 μm particle size; using a UPLC ACQUITY M-Class (Waters, Milford, MA). The mobile phase A consisted of 0.1% formic acid (FA) in water, and the mobile phase B was 0.1% FA in acetonitrile with the following gradient: 0 min 7% B, 30.37 min 40% B, 32.03–35.34 min 85% B, 37–47 min 7% B at a flow of 400 nl/min at 45 °C (column temperature). The spectra data were acquired in a mass spectrometer with electrospray ionization and ion mobility separation Synapt G2-S*i* (Waters, Milford, MA) using a data-independent acquisition approach with a high-definition MSE mode. The ionization was set with the following parameters: 2.75 kV in the sampler capillary, 30 V in the sampling cone, 30 V in the source offset, 70 °C for the source temperature, 0.5 bar for the nanoflow gas, and 150 L/h for the purge gas flow. Two chromatograms were acquired (low and high-energy chromatograms) in a positive mode range of 50–2000 *m/z* with a scan time of 500 ms. No collision energy was applied to obtain the low-energy chromatogram, while for the high-energy chromatograms, the precursor ions were fragmented in the transfer using a collision energy ramp from 19 to 55 eV. All conditions were injected in triplicate, and the Synapt G2-S*i* was calibrated with [Glu1]-fibrinopeptide, [M + 2H]2 + = 785.84261 at 1.5 ppm.

For the analysis, 60 *.raw files containing MS and MS/MS spectra from each fraction were analyzed and quantified label-free with Progenesis QI for Proteomics v4.1(Nonlinear Dynamics, Milford, MA) using a target decoy strategy against the *Mus musculus* *.fasta database (obtained from Uniprot, UP000000589, 55471 protein sequences), which was concatenated with the same *.fasta file in the reverse sense. The parameters used for protein identification were: trypsin as the cutting enzyme and one missed cleavage allowed; carbamidomethyl (C) as a fixed modification and oxidation (M), amidation (C-terminal), deamidation (Q, N) or phosphorylation (S, T, Y) as variable modifications; peptide and fragment tolerance were set to automatic, minimum fragment ion matches per peptide: 2, minimum fragment ion matches per protein: 5, minimum peptide matches per protein: 1, and false discovery rate <4%. The average intensity of the three most abundant peptides per protein (Top3) was used for label-free quantitation according to a previously described method [67]. The Top3 values of a specific protein were summarized if a protein was detected in more than one piece of SDS-PAGE.

All proteins considered differentially expressed (DEPs) in this work displayed at least a fold change (FC) of ±1 (expressed as log_2_), calculated on the Top3 signal of each characterized protein in hypoxia over normoxia. All DEPs have a *p*-value ≤ 0.05, at least two total peptides, including at least one unique peptide. Finally, all DEPs reported in this work were repeated in 3/3 injections.

The mass spectrometry proteomics data have been deposited to the ProteomeXchange Consortium via the PRIDE [42] partner repository with the dataset identifier PXD033915.

### Statistical analysis

GraphPad Prism 9 was used to analyze the data, which were considered significant at a *p* < 0.05. The actual statistical test used for each data set is indicated in the Results section and the Figure legends.

## Supplementary information

Supplemental material

## Data Availability

The proteomic dataset generated and analysed during the current study is available in the ProteomeXchange Consortium via the PRIDE partner repository with the dataset identifier PXD033915. All other datasets generated during and/or analysed during the current study are available from the corresponding author on reasonable request.
