# Supplementary material for: Extracellular vesicles from neural progenitor cells promote functional recovery after stroke in mice with pharmacological inhibition of neurogenesis
Source: Cell Death Discov. 2023 Jul 28;9:272. doi: 10.1038/s41420-023-01561-4 (PMC10382527; doi:10.1038/s41420-023-01561-4)

Supplemental material

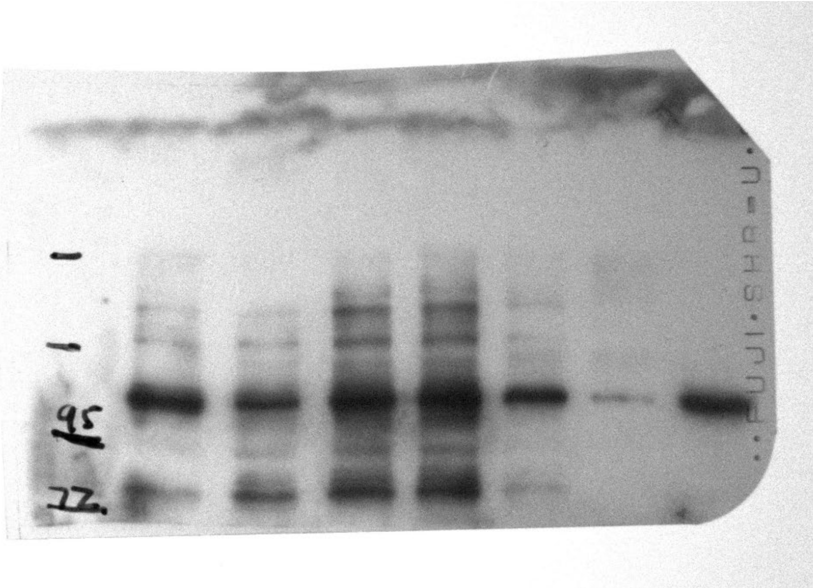

Figure S1. Uncropped WB image of HIF-1. Corresponding to Figure 1F top panel.

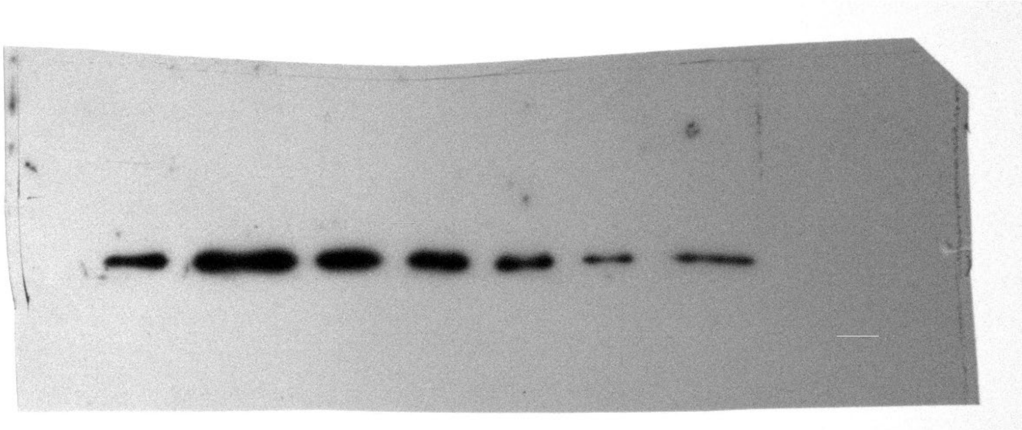

Figure S2. Uncropped WB image of GAPDH. Corresponding to Figure 1 bottom panel.

Uncropped agarose gels from Figure 1G

VEGF

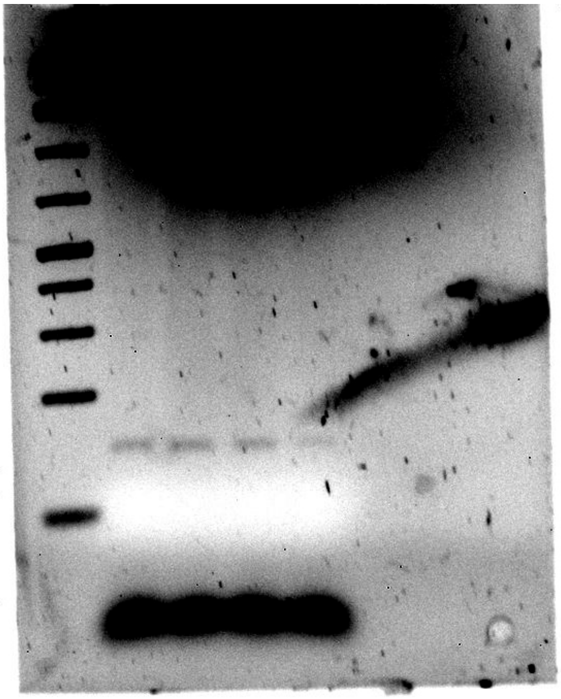

GAPDH

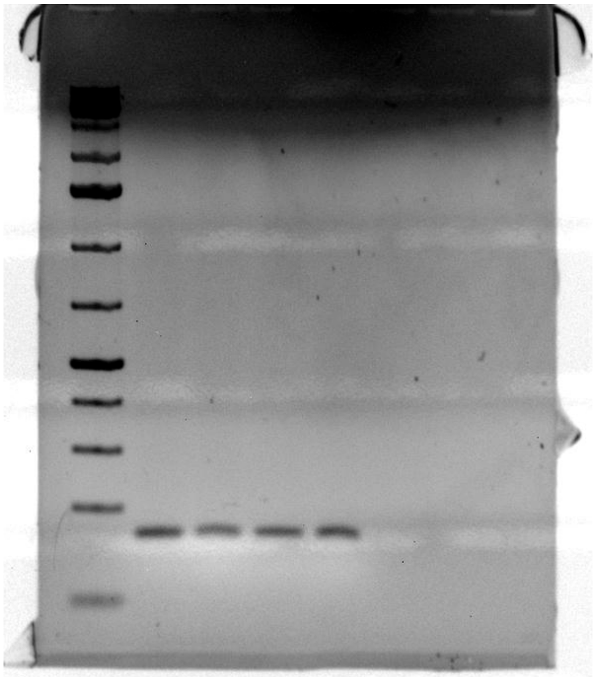

Supplement: Supplementary file 1 — Supplemental material [file 41420_2023_1561_MOESM1_ESM.pdf]
